# Air‐Sea Heat and Moisture Flux Gradients

**DOI:** 10.1029/2024GL110728

**Published:** 2024-11-20

**Authors:** Rhys Parfitt

**Affiliations:** ^1^ Department of Earth, Ocean, and Atmospheric Science Florida State University Tallahassee FL USA

## Abstract

Air‐sea heat and moisture fluxes modulate the surface energy balance and oceanic and atmospheric heat transport across all timescales. Spatial gradients of these fluxes, on a multitude of spatial scales, also have significant impacts on the ocean and atmosphere. Nevertheless, analysis of these gradients, and discussion regarding our ability to represent them, is relatively absent within the community. This letter discusses their importance and presents a wintertime climatology. Their sensitivity to spatiotemporal scale and choice of data set is also examined in the mid‐latitudes. A lead‐lag analysis illustrates that wintertime air‐sea heat flux gradients in the Gulf Stream can precede the North Atlantic Oscillation by ∼1 month. A lack of observations and thus validation of air‐sea heat flux gradients represents a significant gap in our understanding of how air‐sea processes affect weather and climate, and warrants increased attention from the observational and modeling communities.

## Key Points

- Air‐sea heat and moisture flux gradients modulate important oceanic and atmospheric processes across a multitude of spatiotemporal scales
- Air‐sea heat flux gradient variability can statistically precede mid‐latitude atmospheric variability
- Notable air‐sea heat and moisture flux gradient inconsistencies exist in data products, yet the ability to validate them remains elusive

## 1 Introduction

The ocean dominates the zonally integrated poleward heat transport from the tropics into the sub‐tropics and mid‐latitudes (Trenberth & Caron, 2001), where it transfers vast amounts of heat and moisture into the atmosphere via latent and sensible heat fluxes (LHFs and SHFs respectively). These fluxes are estimated through air‐sea differences in the mean “bulk” state variables measured at the surface and at some height within the surface layer (Cronin et al., 2019):

$$\text{LHF}\approx \rho L_{v}C_{E}S∆Q,$$

$$\text{SHF}\approx \rho c_{p}C_{H}S∆\theta ,$$

where ρ is density of air, S is scalar wind speed relative to the ocean surface (including gustiness), $C_{E}$ and $C_{H}$ are drag coefficients for latent and sensible heat respectively, $L_{v}$ is latent heat of evaporation, $c_{p}$ is specific heat at constant pressure, and ∆Q and ∆θ are air‐sea differences in specific humidity and potential temperature respectively.

These heat fluxes are well‐documented in their impact on the atmosphere and ocean, particularly in the wintertime extra‐tropics when LHFs and SHFs are generally much larger due primarily to increased ∆Q and ∆θ (e.g., Yu & Weller, 2007). This is particularly true over western boundary currents (WBCs) in the Northern Hemisphere (NH), where climatological maxima are located due to frequent cold and dry air blowing off large land masses over warm waters. Here, strong air‐sea exchange significantly modulates storm‐development (Cione et al., 1993; Hirata et al., 2019), and anchors vertical motion deep into the troposphere (Minobe et al., 2008). Generally, air‐sea heat fluxes exhibit some of the highest uncertainties of earth system variables in observations and models (e.g., Robertson et al., 2020), and significant efforts are being made to improve their accuracy.

These regions are also characterized by frequent extremely large air‐sea flux gradients (e.g., Parfitt et al., 2016). Such gradients also have an important role in modulating atmospheric and oceanic variability. The exact role likely depends on the spatial scale associated with the gradient. For example, “oceanic baroclinic adjustment” (Hotta & Nakamura, 2011; Nakamura et al., 2004; Taguchi et al., 2009), suggested as a critical component in maintaining baroclinicity in the mid‐latitude storm‐tracks of both the Northern and Southern Hemisphere (SH), is primarily associated with a large‐scale air‐sea heat flux gradient (>100 km). Here, a strong surface air‐temperature gradient formed through differential sensible heat fluxes above an oceanic front favors baroclinic eddy growth (c.f. Figure 12, Sampe et al., 2010). Air‐sea heat flux gradients are also known to impact frontogenesis in the lower atmosphere (Jacobs et al., 2008; Reeder et al., 2021; Tochimoto & Niino, 2022), thus modulating the extra‐tropical transition of tropical cyclones (Jones et al., 2023) and time‐mean structures (Jones et al., 2024; Parfitt & Seo, 2018), with studies indicating fine‐scale gradients (∼25 km) are critical (Parfitt et al., 2016). Furthermore, this effect can overcome data assimilation constraints in reanalysis data sets (Masunaga et al., 2018; Parfitt, Czaja, & Kwon, 2017). Differential air‐sea heat fluxes also modulate oceanic frontogenesis in oceanic frontal regions (Tozuka & Cronin, 2014; Tozuka et al., 2017), with implications for the aforementioned mechanisms, coupled air‐sea feedbacks and climate model evaluation.

Despite the clear importance of air‐sea heat flux gradients, they remain relatively undiscussed in the air‐sea interaction and wider weather and climate communities. For example, there are no comprehensive data set comparisons in air‐sea heat flux gradients, despite many in air‐sea heat fluxes. To the author's knowledge, a published climatology also does not exist. Furthermore, other than rare instances (e.g., Kawai et al., 2015), in virtually all open ocean regions of interest there is no in situ data to validate models and data products for air‐sea heat flux gradients at the aforementioned scales. This is because one requires observations from multiple measurement systems located at a continually consistent distance close (<100 km) to each other. Buoys fitting this criterion are generally located very near the coast (see the National Data Buoy Center for example, https://www.ndbc.noaa.gov/), and individual ship‐based measurements are not suitable for this purpose. Satellite‐derived flux products are also not suitable given that input variables come from different satellites with different overpass times (Gentemann et al., 2020).

The data sets used in this study are introduced in Section 2. In Section 3, a wintertime air‐sea heat flux gradient climatology is considered. A case study is also discussed in the context of atmospheric storms, with attention paid to the spatial scale over which the gradient is calculated. To demonstrate uncertainty, three products are used to conduct a comparison at a location in the Gulf Stream (GS). The relationship at this location of both SHF and the SHF gradient with atmospheric and oceanic variability is also explored. Section 4 presents conclusions and offers potential future directions.

## 2 Data

Air‐sea heat fluxes from three products are used in this study. The first data set used is the European Center for Medium‐Range Weather Forecasts Reanalysis 5 (ERA‐5) product (Hersbach et al., 2020), from 1979 to 2018. ERA5 has a native resolution of ∼0.28° and is provided on a regular 0.25° grid. LHFs and SHFs are provided every hour, and represent an accumulation across the previous hour ending at the validity time. As such, a representative value of LHF (Wm^−2^) at any particular hour t is estimated as $\left(\frac{1}{3600}\right)\left[½\;\left({\text{LHF}}_{t}\right)+½\;\left({\text{LHF}}_{t+1}\right)\right]$.

The second data set used is the Japanese 55‐year Reanalysis (JRA‐55) product (Kobayashi et al., 2015), from 1979 to 2018. JRA‐55 has a native resolution of ∼0.50° and provides all variables on a regular 1.25° grid, which is used here (it is noted that many variables, including LHF and SHF, are also provided on a ∼0.50° grid). In JRA‐55, two datapoints are provided every 6 hr, representing averages of 3‐hr and 6‐hr forecasts from that time. Accordingly, a representative value of LHF (Wm^−2^) at a particular 6‐hourly time t can be estimated from the formula $½\left[\left(2{\text{LHF}}_{t-1}^{6-\text{hour}}-{\text{LHF}}_{t-1}^{3-\text{hour}}\right)+{\text{LHF}}_{t}^{3-\text{hour}}\right]$.

Lastly, a blended product Woods Hole Oceanographic Institution Objectively Analyzed Air‐Sea Fluxes Version 3 (OAFlux‐v3) is used from 1985 to 2018. OAFlux‐v3 uses an integrated analysis method to combine satellite and reanalysis data, and the COARE 3.0 algorithm to calculate heat fluxes (Jin & Weller, 2008). OAFlux‐v3 is provided as daily means on a regular 1° grid.

In addition to LHF and SHF, ERA‐5 is also used for sea‐surface temperature (SST), mean sea‐level pressure (MSLP), as well as 900 hPa air‐temperature (T_900_), zonal wind and meridional wind. Atmospheric frontal systems are calculated from the equation $\frac{\left|{\nabla T}_{900}\right|\zeta_{900}}{f\left|{\nabla T}_{o}\right|}$, where $\zeta_{900}$ is 900 hPa relative vorticity, f is the Coriolis parameter, and $\left|{\nabla T}_{o}\right|$ is a typical temperature gradient 0.45 K/100 km, as in Parfitt, Czaja, and Seo (2017).

## 3 Results

### 3.1 Wintertime Climatology

**Figure 1:**
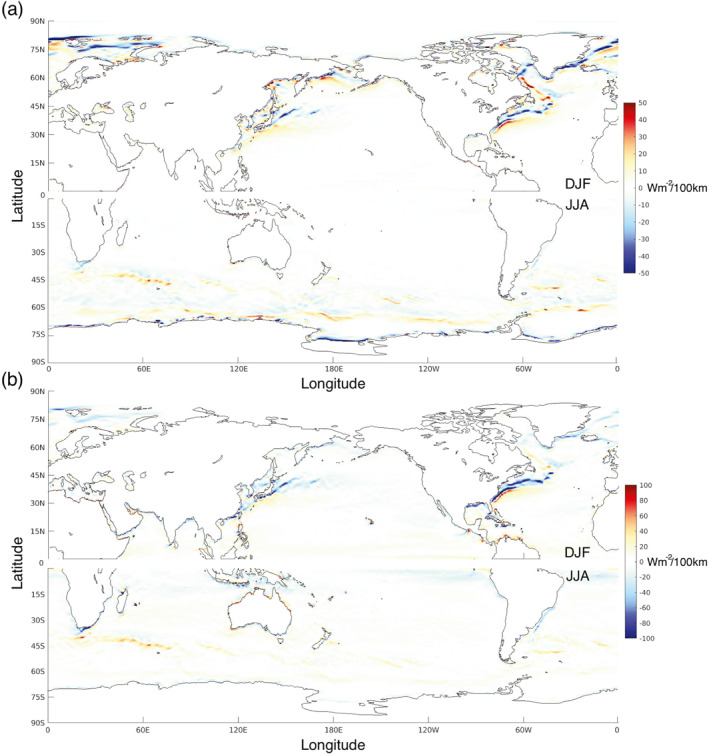
Wintertime (DJF in NH, JJA in SH, 1979–2018) climatology of (a) ${\left(\frac{d\text{SHF}}{dy}\right)}_{∼50\text{km}}$ and (b) ${\left(\frac{d\text{LHF}}{dy}\right)}_{∼50\text{km}}$ in ERA‐5. Positive is defined as northward.

Figure 1a illustrates the meridional SHF gradient climatology in wintertime (December‐February, DJF, in the NH; June‐August, JJA, in the SH). The gradient is calculated at each point in ERA‐5 from the SHF values (hourly, but sampled 6‐hourly) at each neighboring meridional grid point, and as such the scale of the gradient is ∼50 km. For the rest of the paper, this is written as ${\left(\frac{d\text{SHF}}{dy}\right)}_{∼50\text{km}}$, where the temporal resolution is 6‐hourly calculated as described in Section 2 unless specified otherwise. Although the units of all LHF and SHF gradients in this manuscript are given in Wm^−2^/100 km, the scale over which the gradient is calculated is important and will be specified. It is noted that the meridional SHF gradient is illustrated instead of the absolute gradient magnitude to retain the sign, which is important for the associated air‐sea interaction processes (Parfitt et al., 2016). The meridional temperature gradient is also most relevant for baroclinity (Walland & Simmonds, 1999), and hence mid‐latitude storm development in general. For reference, zonal gradients are shown in Figure S1 in Supporting Information S1.

In the NH, the largest average ${\left(\frac{d\text{SHF}}{dy}\right)}_{∼50\text{km}}$ magnitudes (∼50 Wm^−2^/100 km) are typically in regions associated with large meridional SST gradients. In the North Atlantic, this is across the GS, the Labrador Current, and the Greenland Current. In the North Pacific, this is in the Sea of Japan, across the Kuroshio‐Oyashio Current system, and in the north of the Bering Sea. This is also true in the SH, with large average ${\left(\frac{d\text{SHF}}{dy}\right)}_{∼50\text{km}}$ magnitudes found at the Agulhas Return Current and Brazil‐Malvinas Confluence Region, as well as oceanic frontal zones along the Antarctic Circumpolar Current, although they are generally smaller than their NH counterparts. It is noted that the largest average ${\left(\frac{d\text{SHF}}{dy}\right)}_{∼50\text{km}}$ magnitudes, however, are comparable and are found near the sea‐ice margin off the coast of Antarctica.

Figure 1b illustrates the analogous wintertime ${\left(\frac{d\text{LHF}}{dy}\right)}_{∼50\text{km}}$ climatology. There is a high degree of similarity in spatial structure with Figure 1a, with the largest average ${\left(\frac{d\text{LHF}}{dy}\right)}_{∼50\text{km}}$ corresponding to regions of high meridional SST gradients. Across the GS and Kuroshio‐Oyashio systems however, the average magnitude is almost double that of ${\left(\frac{d\text{SHF}}{dy}\right)}_{∼50\text{km}}$, reaching ∼100 Wm^−2^/100 km, while off the coast of Antarctica the average magnitude is greatly reduced. The presence of wintertime average ${\left(\frac{d\text{LHF}}{dy}\right)}_{∼50\text{km}}$ magnitudes up to ∼20 Wm^−2^/100 km are also noted in the Tropics.

Recent studies suggest the ocean is the primary driver of co‐variability between mid‐latitude SSTs and air‐sea heat and moisture fluxes at the oceanic mesoscale (Bishop et al., 2017; Small et al., 2019). It is thus not surprising that in the mid‐latitudes, the structure of ${\left(\frac{d\text{SHF}}{dy}\right)}_{∼50\text{km}}$ and ${\left(\frac{d\text{LHF}}{dy}\right)}_{∼50\text{km}}$ variability closely mirrors that of meridional SST gradients. In general, climatological ${\left(\frac{d\text{SHF}}{dy}\right)}_{∼50\text{km}}$ and ${\left(\frac{d\text{LHF}}{dy}\right)}_{∼50\text{km}}$ act to enhance (reduce) the climatological meridional atmospheric temperature gradients in both the NH and SH on the poleward (equatorward) side of warm currents such as the mid‐latitude frontal zones, suggesting variability in latitude of these zones will likely influence atmospheric variability (Joyce et al., 2019).

### 3.2 Case Study and Importance of Scale

**Figure 2:**
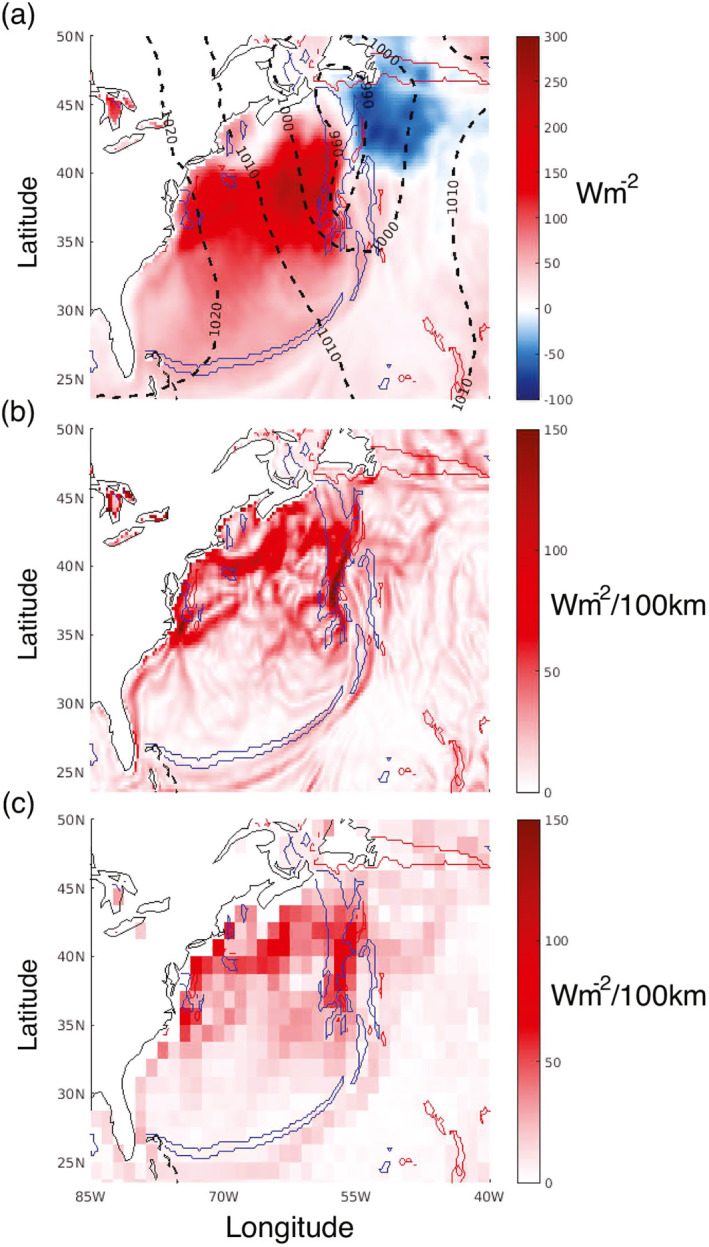
An extra‐tropical cyclone in the North Atlantic identified in ERA‐5 at 1200UTC 20 January 1979. (a) SHF (color, positive defined as ocean to atmosphere), mean sea‐level pressure (dotted black contours), and atmospheric cold and warm fronts (blue and red contours). (b) ${|\nabla \text{SHF}|}_{∼50\text{km}}$, with atmospheric fronts overlaid. (c) ${|\nabla \text{SHF}|}_{∼250\text{km}}$, with atmospheric fronts overlaid.

As discussed in the introduction, LHF and SHF gradients in the “hotspot” regions in Figure 1, such as WBCs, can impact weather systems both through direct influence and pre‐conditioning. The gradient spatial scale, however, is critically important to the interaction. Figure 2 considers a case study of an extra‐tropical cyclone across the GS on 1200UTC 20 January 1979 in ERA‐5. Figure 2a illustrates the MSLP (dotted contours) and atmospheric cold (warm) fronts at 900 hPa in blue (red) contours. As expected, a broad region of strong ocean‐to‐atmosphere (atmosphere‐to‐ocean) SHF exists in the cold (warm) sector, associated with the air mass influence on ∆θ. At first glance, the SHFs in each sector appear relatively homogeneous. However, analysis of ${|\nabla \text{SHF}|}_{∼50\text{km}}$ in Figure 2b illustrates fine‐scale underlying structure, with the strongest ${|\nabla \text{SHF}|}_{∼50\text{km}}$ located both in the cold sector and across the cold‐front north of ∼37.5 N, likely associated with co‐located sharp SST and atmospheric temperature gradients. It is noted that larger ${|\nabla \text{SHF}|}_{∼50\text{km}}$ are not always exactly co‐located with the 900 hPa cold front due to cold frontal tilt with height. Such ${|\nabla \text{SHF}|}_{∼50\text{km}}$ are expected to strongly modulate the atmospheric temperature gradients within the storm.

Figure 2c illustrates the same as Figure 2b, however for a different gradient spatial scale, ${|\nabla \text{SHF}|}_{∼250\text{km}}$—that is, the SHF gradient magnitude is calculated using neighboring grid points 1.25° away, instead of 0.25°. The resulting magnitudes are much smaller everywhere; in weather or climate modeling experiments at these resolutions, there will therefore be noticeable impacts on the associated air‐sea interaction processes. Additionally, while the spatial structure is naturally much coarser, there are areas (e.g., just after GS separation at ∼35 N) where the structure is significantly different. The disparity between the two spatial scales can also be seen for $\frac{d\text{SHF}}{dy}$ and $\frac{d\text{SHF}}{dx}$ (Figure S2 in Supporting Information S1). Indeed, many modeling studies in past decades have considered the impact of smoothed/coarser WBC SST gradients on mid‐latitude storm‐tracks, yet have often arrived at differing conclusions (e.g., Booth et al., 2012; Brayshaw et al., 2011; De Vries et al., 2019; Woollings et al., 2010). Indeed, the question regarding the extent to which SST gradients impact the mid‐latitude atmosphere is still relatively open (Seo et al., 2023). Much of this disagreement may result from a lack of consistency in the smoothing methodology, either in region considered, method employed, or SST data set used, and indirectly the spatial scales at which the SST gradient is smoothed.

### 3.3 Product Comparison

To further discussion on the importance of specificity when calculating SHF and LHF gradients, distributions on various scales and in various different products are considered at (70 W, 37.5 N) in the GS for a common period DJF 1985–2018. Three products are considered, OAFlux‐v3 (1°, daily means), JRA‐55 (1.25°, 6‐hourly) and ERA‐5 (0.25°, hourly sampled every 6‐hr).

**Figure 3:**
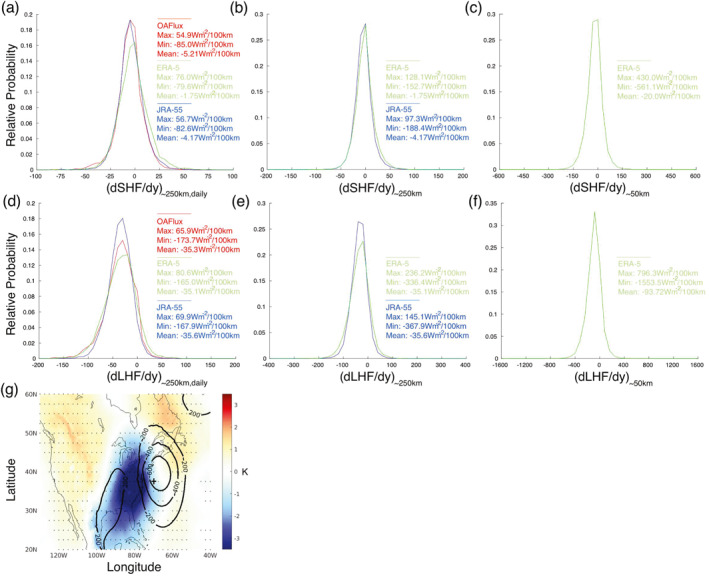
(a) Distribution of ${\left(\frac{d\text{SHF}}{dy}\right)}_{∼250\text{km},\text{daily}}$ at 70 W, 37.5 N (Wm^−2^/100 km) for DJF 1985–2018 in OAFlux‐v3, JRA‐55 and ERA‐5. (b) As in panel (a), but for ${\left(\frac{d\text{SHF}}{dy}\right)}_{∼250\text{km}}$ in JRA‐55 (6‐hourly) and ERA‐5 (hourly, sampled 6‐hourly). (c) As in panel (a), but for ${\left(\frac{d\text{SHF}}{dy}\right)}_{∼50\text{km}}$ in ERA‐5 (hourly, sampled 6‐hourly). (d)–(f) As in panels (a–c) respectively, but for $\frac{d\text{LHF}}{dy}$. (g) 900 hPa temperature anomalies in color (dotted statistical significance to 95%), with contoured mean sea‐level pressure anomalies, composited on times when ${\left(\frac{d\text{SHF}}{dy}\right)}_{∼50\text{km}}$ <−100 Wm^−2^/100 km at 70 W, 37.5 N (marked with a cross).

Firstly, SHF and LHF in OAFlux‐v3 and ERA‐5 are interpolated to the JRA‐55 grid. Then, daily averages are calculated in JRA‐55 and ERA‐5 from values at 6‐hourly intervals (0000, 0600, 1200, 1800 UTC, calculated as described in Section 2). ${\left(\frac{d\text{SHF}}{dy}\right)}_{∼250\text{km},\text{daily}}$ and ${\left(\frac{d\text{LHF}}{dy}\right)}_{∼250\text{km},\text{daily}}$ are then calculated every day at (70 W, 37.5 N) from these daily means, and the probability distributions are plotted in Figures 3a and 3d respectively. For ${\left(\frac{d\text{SHF}}{dy}\right)}_{∼250\text{km},\text{daily}}$, mean values for all three products are relatively close to zero, although there is significant variability across both positive and negative values (maximum and minimum values are also provided for reference). For values close to zero, JRA‐55 and OAFlux‐v3 are in good agreement, however notable divergence begins to occur as the absolute magnitudes increase. The distribution of values in ERA‐5 are weighted much more heavily to larger positive magnitudes. For ${\left(\frac{d\text{LHF}}{dy}\right)}_{∼250\text{km},\text{daily}}$, despite the mean values in each product being in good agreement, the distributions in all products exhibit much more disagreement than for ${\left(\frac{d\text{SHF}}{dy}\right)}_{∼250\text{km},\text{daily}}$ across all values. Again, the distribution of values in ERA‐5 are weighted the most heavily to larger absolute magnitudes.

Next, SHF and LHF values in JRA‐55 and ERA‐5 are used at 6‐hourly intervals to calculate distributions of ${\left(\frac{d\text{SHF}}{dy}\right)}_{∼250\text{km}}$ and ${\left(\frac{d\text{LHF}}{dy}\right)}_{∼250\text{km}}$ at the same location (Figures 3b and 3e respectively). As expected, by removing daily temporal averaging, the mean values stay the same but distribution spread increases. In particular, absolute magnitudes of ${\left(\frac{d\text{SHF}}{dy}\right)}_{∼250\text{km}}$ above 50Wm^−2^/100 km and ${\left(\frac{d\text{LHF}}{dy}\right)}_{∼250\text{km}}$ above 100Wm^−2^/100 km are observed more frequently than in the daily means, suggesting ${\left(\frac{d\text{SHF}}{dy}\right)}_{∼250\text{km}}$ and ${\left(\frac{d\text{LHF}}{dy}\right)}_{∼250km}$ of these magnitudes typically persist here less than a day. Similar to the daily means, ${\left(\frac{d\text{SHF}}{dy}\right)}_{∼250\text{km}}$ and ${\left(\frac{d\text{LHF}}{dy}\right)}_{∼250\text{km}}$ distributions in ERA‐5 generally exhibit fatter tails than those in JRA‐55.

Lastly, SHF and LHF values in ERA‐5 are used at 6‐hourly intervals, on their original 0.25° grid to calculate distributions of ${\left(\frac{d\text{SHF}}{dy}\right)}_{∼50\text{km}}$ and ${\left(\frac{d\text{LHF}}{dy}\right)}_{∼50\text{km}}$ at the same location (Figures 3c and 3f respectively), with the units still in Wm^−2^/100 km. At these spatial scales, the absolute magnitude of the mean increases noticeably. Furthermore, values over three to four times larger are found in ${\left(\frac{d\text{SHF}}{dy}\right)}_{∼50\text{km}}$ and ${\left(\frac{d\text{LHF}}{dy}\right)}_{∼50\text{km}}$ than in ${\left(\frac{d\text{SHF}}{dy}\right)}_{∼250\text{km}}$ and ${\left(\frac{d\text{LHF}}{dy}\right)}_{∼250\text{km}}$, indicative of significant finer‐scale variability at this location that would not be captured in a data set of lower spatial resolution. Figure 3g illustrates atmospheric 900 hPa temperature anomalies in color (dotted statistical significance to 95%), with contoured MSLP anomalies, composited on times when ${\left(\frac{d\text{SHF}}{dy}\right)}_{∼50\text{km}}$ < −100 Wm^−2^/100 km at (70 W, 37.5 N). These ${\left(\frac{d\text{SHF}}{dy}\right)}_{∼50\text{km}}$ magnitudes not found at ∼250 km scales are typically associated with sharp atmospheric temperature gradients embedded within extra‐tropical cyclones, that is, atmospheric fronts.

### 3.4 Influence of Air‐Sea Flux Gradients on the North Atlantic Oscillation

**Figure 4:**
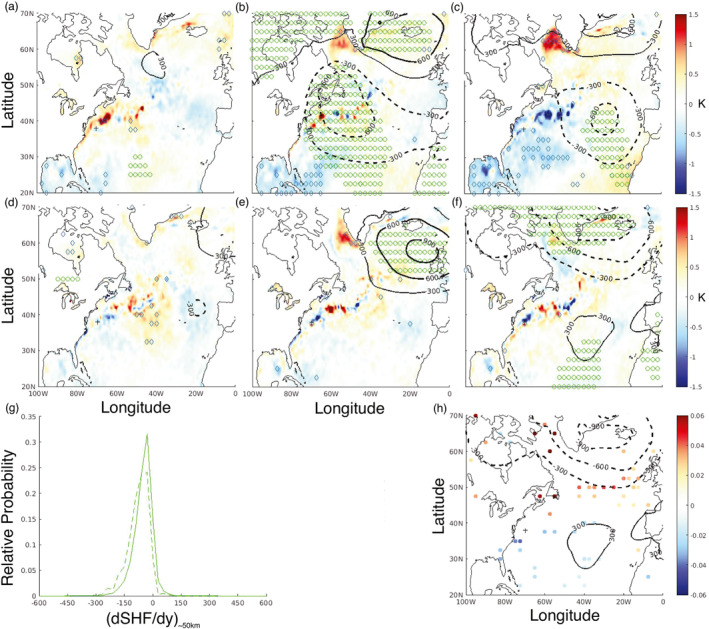
Monthly (a) December (−1 month) (b) January (simultaneous) (c) February (+1 month) anomalies in sea‐surface temperature (SST) (color) and mean sea‐level pressure (MSLP) (contours), composited on the 10% most positive January monthly mean values of SHF at 70 W, 38 N during the period DJF 1979–2018. Statistical significance to 90% shown for SST (blue diamonds) and MSLP (green circles). (d–f) As in panels (a–c) except composited on the 10% most negative January monthly mean values of ${\left(\frac{d\text{SHF}}{dy}\right)}_{∼50\text{km}}$. (g) Distribution of ${\left(\frac{d\text{SHF}}{dy}\right)}_{∼50\text{km}}$ at 70 W, 38 N (Wm^−2^/100 km) for the DJF seasons used in the panels (d–f) composites (dotted green), as well as for DJF 1979–2018 (solid green). (h) As in panels (f) but with atmospheric cold frontal anomalies, where significant to 95%, plotted as colored circles.

Traditionally, extra‐tropical air‐sea heat fluxes are viewed as being driven mainly by atmospheric variability. For example, the leading monthly mode of North Atlantic SST variability has been extensively noted to reflect variability in the North Atlantic Oscillation (NAO) and subsequent atmospheric forcing of the air‐sea heat fluxes (c.f. Figure 2, Marshall et al., 2001). Figure 4 shows the ERA‐5 lead‐lag composite [(a) −1 month, December, (b) 0 lag, January, and (c) +1 month, February] of both monthly SST (color) and MSLP (contours) anomalies, composited on the 10% most positive (i.e., ocean‐to‐atmosphere) monthly January‐mean SHF values at 70 W, 38 N between 1979 and 2018. Significance as measured by a two‐sample *t*‐test to 90% is shown as blue diamonds (green circles) for SST (MSLP). Strong monthly January SHF is associated with strong co‐located anomalous low‐pressure (Parfitt & Czaja, 2016; Zolina & Gulev, 2003), appearing as part of the basin‐wide NAO‐negative signal. In February, the basin‐wide MSLP signal dissipates and significant SST anomalies become more widespread. Although the composite sample size is small, the NAO‐SHF relationship can also be seen in a 39‐season correlation of anomalous monthly January SHF at this location with the monthly NAO index (Hurrell et al., 2003) in January (*r* = −0.40, *p* = 0.01) and February (negligible).

The same procedure as in Figures 4a–4c is now applied to the most negative 10% of monthly mean January ${\left(\frac{d\text{SHF}}{dy}\right)}_{∼50\text{km}}$ values in Figures 4d–4f. There is little basin‐wide signal in either SST or MSLP at zero lag (although anomalous high‐pressure exists to the east of Greenland, and a statistically significant SST anomaly at the location itself). Instead, a basin‐wide NAO‐type MSLP (but not SST) anomaly is found in February. Indeed, the analogous 39‐season correlation between anomalous monthly January ${\left(\frac{d\text{SHF}}{dy}\right)}_{∼50\text{km}}$ at this location and the monthly January NAO index is negligible, but weakly significant 1 month later for the February NAO (*r* = −0.28, *p* = 0.08). Neighboring grid‐points more strongly exhibit this relationship between anomalous monthly January ${\left(\frac{d\text{SHF}}{dy}\right)}_{∼50\text{km}}$ and the February NAO index (e.g., 70 W, 38.25 N: *r* = −0.33, *p* = 0.04; 70 W, 38.5 N: *r* = −0.31, *p* = 0.05). However, the grid‐point used in Figure 3 does not (70 W, 37.5 N), nor does point (70 W, 38.75 N); these points have an average ${\left(\frac{d\text{SHF}}{dy}\right)}_{∼50\text{km}}$ considerably closer to zero than the points exhibiting the relationship, likely due to increased distance from the average GS frontal axis. It is worth noting that a data set with values every 1.25° would not pick up the three points of significance discussed above.

The intention here is simply to illustrate how air‐sea flux gradients provide information separate from air‐sea fluxes alone, with detailed dynamical exploration left for a companion study. However, Parfitt and Kwon (2020) discussed how an enhanced ${\left(\frac{d\text{SHF}}{dy}\right)}_{∼50\text{km}}$ (note this equates to more negative values at this location) can impact diabatic frontogenesis and potentially the eddy‐driven jet latitude, offering one potential explanation for the relationship in Figure 4f. Figure 4g shows the DJF distribution of ${\left(\frac{d\text{SHF}}{dy}\right)}_{∼50\text{km}}$ for the four seasons used in Figure 4f and demonstrates that significant negative ${\left(\frac{d\text{SHF}}{dy}\right)}_{∼50\text{km}}$ are much more common than in typical DJF seasons. Figure 4h illustrates the same as Figure 4f, except instead of SST, monthly atmospheric cold frontal anomalies are plotted as dots where they are significant to 95%. The units are of absolute fractional frequency (i.e., 0.06 would mean a cold front is identified here, say, 14% of the time in February vs. a February average of 8%). Comparison with typical atmospheric frontal climatologies (e.g., Berry et al., 2011; Soster & Parfitt, 2022) show these anomalies reach percentage changes of ∼50%, and are consistent with the MSLP anomalies (i.e., more atmospheric cold fronts with lower MSLP and vice versa).

## 4 Discussion

Despite several studies illustrating the importance of SHF and LHF gradients, the statistics of their variability, their representation in data products, and their overall influence on weather and climate are rarely discussed in the literature. This study presents a wintertime climatology of meridional SHF and LHF gradients at the ∼50 km scale in ERA‐5. Generally, large climatological magnitudes are found at oceanic frontal zones, regions with large SST gradients. This is unsurprising, given that mesoscale SHF and LHF fluxes have recently been argued as ocean‐driven. In reanalyses and models this is significant (e.g., Kirtman et al., 2012), and only recently have we been able to represent such fine‐scale heat fluxes—for example, the average SST resolution in the Coupled Model Intercomparison Project 5 (Taylor et al., 2012) was only ∼1°.

Often, air‐sea interaction studies considering gradients do not specify the scales that are being considered, potentially leading to inconsistency between conclusions (a well‐known instance is the importance of the GS SST gradient for North Atlantic variability). A case study of ${|\nabla \text{SHF}|}_{∼50\text{km}}$ and ${|\nabla \text{SHF}|}_{∼250\text{km}}$ within an extra‐tropical cyclone demonstrates clear differences ‐ a model experiment at these different resolutions, or with SST smoothing at these different scales, will impact the atmospheric system differently. Studies have also shown explicitly that GS gradient variability at ∼50km is different from that at larger scales (Parfitt et al., 2022), and modeling studies to further ascertain the sensitivity of ocean‐weather interactions to different scales in |∇SHF| and |∇LHF| would be useful.

Significant differences between three different data products are illustrated across the GS in distributions of ${\left(\frac{d\text{SHF}}{dy}\right)}_{∼250\text{km},\text{daily}}$, ${\left(\frac{d\text{SHF}}{dy}\right)}_{∼250\text{km}}$ and ${\left(\frac{d\text{SHF}}{dy}\right)}_{∼50\text{km}}$. A notable issue is that there are almost no corresponding in situ observations to validate these products in such regions (e.g., buoys that remain a constant distance close to each other). The fact that large disagreements primarily exist in the distribution tails, often induced by fast‐moving atmospheric fronts, also means that satellite‐derived flux products cannot serve as a validation tool either. This is because input variables come from different satellites with different overpass times (Gentemann et al., 2020); for example, the product Institut Français pour la Recherche et l’Exploitation de la Mer (Bentamy et al., 2013) bins observations in a 3‐hr window, whereas the Japanese Ocean Flux Data Sets with Use of Remote Sensing Observations product (Tomita et al., 2019) bins over an entire day.

Lastly, an example is shown of monthly variability in GS ${\left(\frac{d\text{SHF}}{dy}\right)}_{∼50\text{km}}$ statistically preceding the NAO by a month, but with high sensitivity to the exact location (to < 100km). Such results provide clear evidence of the need to expand in situ networks (e.g., Clayson et al., 2021; Diard et al., 2019), especially away from coastlines, and improve satellite‐derived products. Future open ocean observational deployments should include methodologies (e.g., multiple in situ platforms, Farrar et al., 2020; in‐tandem uncrewed surface vehicles, Cronin et al., 2024) for maintaining air‐sea flux measurements at consistent distances (in particular < 250km) from each other for extended periods.

## Supporting information

Supporting Information S1

## Data Availability

ERA5 (Hersbach et al., 2023) data can be freely obtained from the Climate Data Store. JRA‐55 (Japan Meteorological Agency, 2013) data can be freely obtained from the Research Data Archive, as can OAFlux v3 (Goddard Institute for Space Studies et al., 2006). The NAO index (Hurrell et al., 2023) is available from the Climate Data Guide.
