# Supplementary material for: Air‐Sea Heat and Moisture Flux Gradients
Source: Geophys Res Lett. 2024 Nov 20;51(22):e2024GL110728. doi: 10.1029/2024GL110728 (PMC11578016; doi:10.1029/2024GL110728)
Supplement: Supplementary file 1 — Supporting Information S1 [file GRL-51-0-s001.pdf]

Geophysical Research Letters

Supporting Information for

Air-Sea Heat and Moisture Flux Gradients

Rhys Parfitt

Department of Earth, Ocean, and Atmospheric Science, Florida State University, Tallahassee, Florida, USA

Contents of this file

Figures S1 to S2

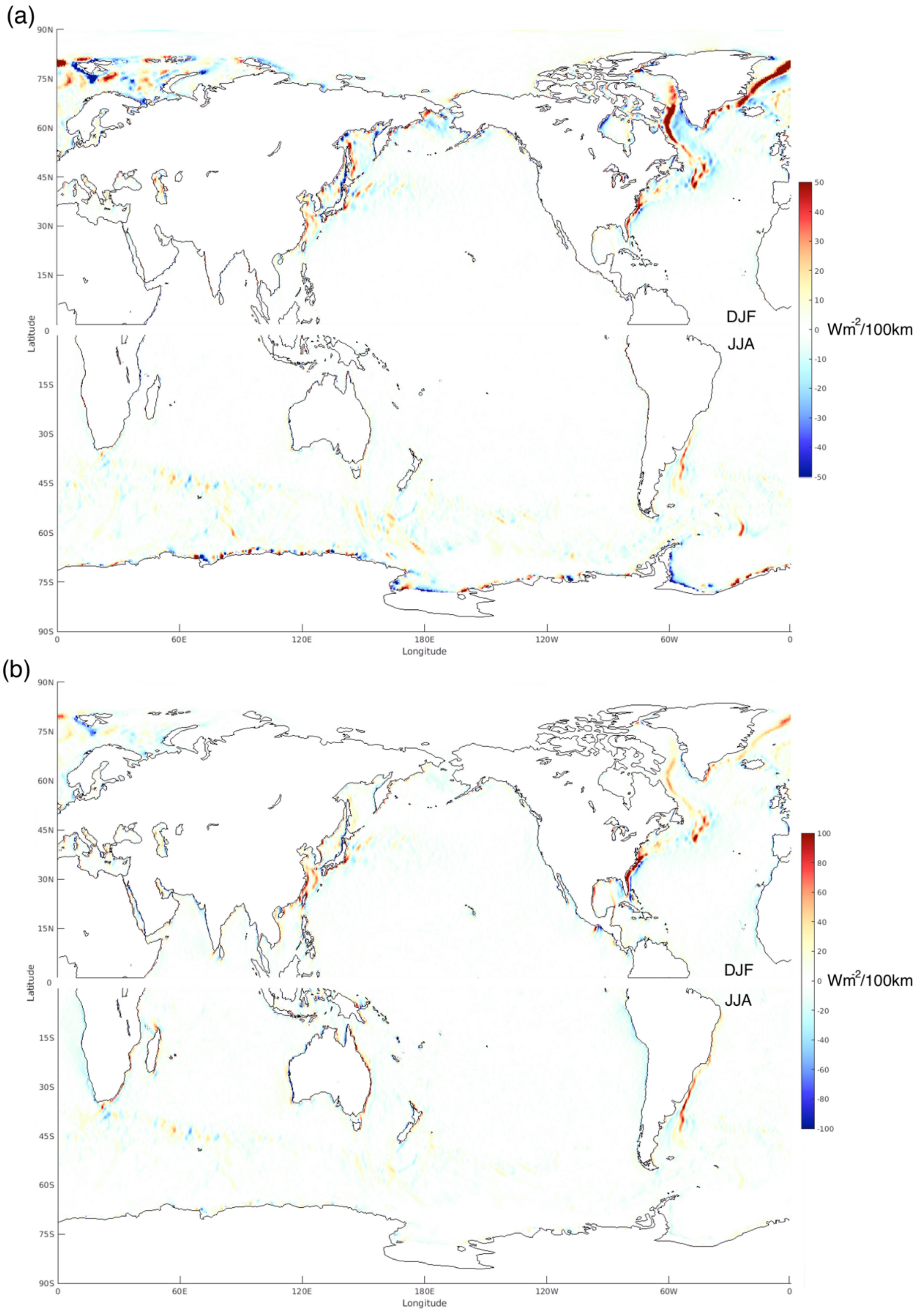

Figure S1: Wintertime (DJF in NH, JJA in SH, 1979-2018) climatology of (a) $(\frac{dSHF}{dx})_{\sim 50km}$ and (b) $(\frac{dLHF}{dx})_{\sim 50km}$ in ERA-5. Positive is defined as eastward.

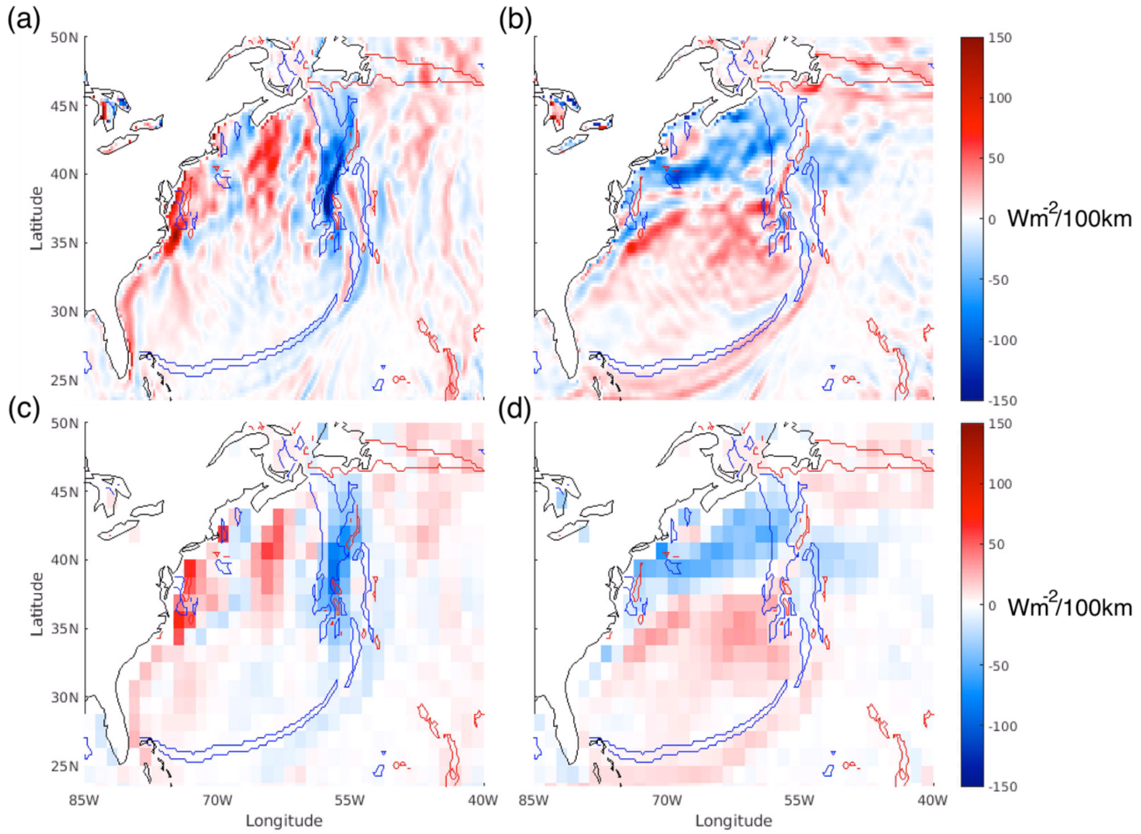

Figure S2: An extra-tropical cyclone in the North Atlantic identified in ERA-5 at 1200UTC 20th January 1979. (a) $(\frac{dSHF}{dx})_{\sim 50km}$, with atmospheric cold and warm fronts overlaid (blue and red contours), (b) $(\frac{dSHF}{dy})_{\sim 50km}$, (c) $(\frac{dSHF}{dx})_{\sim 250km}$, (d) $(\frac{dSHF}{dy})_{\sim 250km}$.
